# Community participation in health services development, implementation, and evaluation: A systematic review of empowerment, health, community, and process outcomes

**DOI:** 10.1371/journal.pone.0216112

**Published:** 2019-05-10

**Authors:** Victoria Haldane, Fiona L. H. Chuah, Aastha Srivastava, Shweta R. Singh, Gerald C. H. Koh, Chia Kee Seng, Helena Legido-Quigley

**Affiliations:** 1 Swee Hock School of Public Health, National University of Singapore, Singapore; 2 Department of Global Health and Development, London School of Hygiene and Tropical Medicine, London, United Kingdom; Johns Hopkins School of Public Health, UNITED STATES

## Abstract

**Background:**

Community participation is widely believed to be beneficial to the development, implementation and evaluation of health services. However, many challenges to successful and sustainable community involvement remain. Importantly, there is little evidence on the effect of community participation in terms of outcomes at both the community and individual level. Our systematic review seeks to examine the evidence on outcomes of community participation in high and upper-middle income countries.

**Methods and findings:**

This review was developed according to PRISMA guidelines. Eligible studies included those that involved the community, service users, consumers, households, patients, public and their representatives in the development, implementation, and evaluation of health services, policy or interventions. We searched the following databases from January 2000 to September 2016: Medline, Embase, Global Health, Scopus, and LILACs. We independently screened articles for inclusion, conducted data extraction, and assessed studies for risk of bias. No language restrictions were made. 27,232 records were identified, with 23,468 after removal of duplicates. Following titles and abstracts screening, 49 met the inclusion criteria for this review. A narrative synthesis of the findings was conducted. Outcomes were categorised as process outcomes, community outcomes, health outcomes, empowerment and stakeholder perspectives. Our review reports a breadth of evidence that community involvement has a positive impact on health, particularly when substantiated by strong organisational and community processes. This is in line with the notion that participatory approaches and positive outcomes including community empowerment and health improvements do not occur in a linear progression, but instead consists of complex processes influenced by an array of social and cultural factors.

**Conclusion:**

This review adds to the evidence base supporting the effectiveness of community participation in yielding positive outcomes at the organizational, community and individual level.

**Trial registration:**

**Prospero record number:** CRD42016048244.

## Introduction

Community participation came to the fore with the 1978 Alma Ata declaration, which framed the community as central to the planning, organizing, operation and control of primary health care [1]. In recent years, community participation has once again emerged as a priority in health globally following the initiation of the new Sustainable Development Goals. In line with the SDGs, integrated people-centered health services are key to achieving universal health coverage and attaining this goal requires participatory approaches [2]. Furthermore, with the rapid increase of chronic disease burden worldwide, intersectoral approaches encompassing community participation and engagement has been identified as key for implementing strategies in health promotion and the prevention and control of chronic diseases [3].

Over the decades, there has been much exploration, development, and debate on ways to conceptualize meaningful community participation in health services[4]. Beyond the use of community participatory approaches to promote the effectiveness of health programs implemented, engaging communities effectively is believed to have a positive impact on social capital, leading to enhanced community empowerment, and ultimately improved health status and reduced health inequalities [5]. However, despite the wide acceptance of community involvement in theory and practice, there still remains many challenges, both structural and practical, to successful implementation [5]. Furthermore, there is little concrete evidence on the effectiveness of community involvement programs, particularly on improvements in intermediate and long-term outcomes, including health related outcomes [6]. Much of the research done on community participation has also focused on low and middle income countries despite evidence of its universal utility in improving health [7]. To address this gap, this systematic review aims to examine the evidence on community involvement and participation from studies that report on program outcomes in high and upper-middle income countries.

Previous systematic reviews of community participation outcomes have focused on mother and child health [2], and rural health [8]. One systematic review explored health and social outcomes of participatory approaches in the United Kingdom [9], and one systematic review of literature between 1966 to 2000 reported on the effects of involving patients in the planning and development of healthcare [10]. To our knowledge, there are no reviews of the existing systematic approaches that examine outcomes of community involvement in health service planning, implementation, monitoring, and evaluation for a variety of diseases in high and upper-middle income countries. This review seeks to fill this knowledge gap.

## Methods

This review was developed according to PRISMA guidelines (see S1 Table) [11] and submitted to Prospero at study initiation under record number CRD42016048244. Drawing on the definitions by George et al. (2015)[12], the concept of community and community participation is described in Box 1.

**Box 1. Definitions**

Community: Communities are defined as constituted by those with a shared social identity; that is of members of the same set of social representations, which are the meanings, symbols, and aspirations through which people make sense of their world.

Community participation: Active group participation or participation of a person as representative of the group in activities where they not only provide ideas but are also involved in the intervention.

### Data sources

We developed the search string in accordance with the underlying objective of the study and refined it with inputs from an information specialist. The following databases were searched from January 2000 to September 2016: Medline, Global Health, Embase, Scopus, and LILACs. The full search terms used for Medline are shown in Table 1.

**Table 1:** Medline search string.

Conceptual Areas	MeSH terms and free text terms
Community/patient/consumer participation or engagement	“Community Networks” [MeSH] OR “communit*” [keyword] “community based organizations” [keyword] OR “Community representatives” [keyword] OR “Community leaders” [keyword]OR “Community health workers” [MeSH] OR “Community Involvement” [keyword] or “Community-Institutional Relations” [MeSH] OR “Community based Participatory work” [MeSH] OR “Consumer participation” [MeSH] OR “community participation” [keyword] OR “Communit* Involvement” [keyword] OR “Communit*Engag*” [keyword] OR “community mobilization” [keyword] OR “Communit* representation” [keyword] OR “participatory action research” [keyword] or “Social Participation” [MeSH] OR “Community participants” [keyword] “area participants” [keyword] or “sector participants” [keyword] or “neighbourhood participants” [keyword] or “citizen participants” [keyword]
Intervention in planning/ implementation/monitoring and evaluation	“Health Planning” [MeSH] OR “Community Health Planning” [MeSH] OR “supply chain management” [keyword] OR “Health plan implementation” [MeSH] OR “Outcome and Process Assessment” [MeSH] OR “Program Evaluation” [MeSH] OR “program development” [keyword] OR “program monitoring” [keyword] OR “process monitoring” [keyword] OR “process evaluation” [keyword] OR “Outcome Assessment (Health Care)” [MeSH] OR “Public Health Practice” OR “Hospital Planning” [MeSH]
Outcomes/ capacity-building	“Capacity Building” [MeSH] OR “Health Policy” [MeSH] OR “Quality of Life” [MeSH] OR “Health Services Accessibility”[MeSH] OR “Improved health” [keyword] OR “Delivery of health care” [MeSH] OR “Community health services” [MeSH] OR ‘Patient Acceptance of Health Care" [MeSH] OR “Patient Satisfaction” [MeSH] OR “help-seeking” [keyword] OR “power relations” [keyword] OR “power sharing” [keyword] OR “Attitude to Health” [MeSH] OR “Policy Making” [MeSH] OR “Health Care reform” [MeSH] OR ‘Health Promotion” [MeSH] OR “Health Behavior” [MeSH] OR “Health Status” [MeSH] OR “Health Education” [MeSH] OR “Dissent and Disputes” [keyword]
High income and upper-middle income countries	“Argentina” OR “Albania” OR “Fiji” OR “Namibia” OR “Algeria” OR “Gabon” OR “Palau” OR “American Samoa” OR “Georgia” OR “Panama” OR “Angola”OR “Grenada” OR “Paraguay” OR “Azerbaijan” OR “Guyana” OR “Peru”OR “Belarus” OR “Iran” OR “Romania” OR “Belize” OR “Iraq” OR “Russian Federation” OR “Bosnia and Herzegovina” OR “Jamaica” OR “Serbia” OR “Botswana” OR “Jordan” OR “South Africa” OR “Brazil” OR “Kazakhstan” OR “St. Lucia” OR “Bulgaria” OR “Lebanon” OR “St. Vincent and the Grenadines” OR “China” OR “Libya” OR “Suriname” OR “Colombia’ OR “Macedonia” OR “Thailand” OR ‘Costa Rica” OR “Malaysia” OR “Turkey” OR “Cuba” OR “Maldives” OR “Turkmenistan” OR “Dominica” OR “Marshall Islands” OR “Tuvalu” OR “Dominican Republic” OR “Mauritius” OR “Venezuela” OR “Guinea” OR “Mexico” OR “Ecuador” OR “Montenegro”OR “Andorra” OR “Gibraltar” OR “Oman” OR “Antigua and Barbuda” OR “Greece” OR “Poland” OR “Aruba” OR “Greenland” OR “Portugal” OR “Australia” OR “Guam” OR “Puerto Rico” OR “Austria” OR “Hong Kong” OR “Qatar” OR “Bahamas” OR “Hungary” OR “San Marino” OR “Bahrain” OR “Iceland” OR “Saudi Arabia” OR “Barbados” OR “Ireland” OR “Seychelles” OR “Belgium” OR “Isle of Man” OR “Singapore” OR “Bermuda” OR “Israel” OR “Sint Maarten” OR “British Virgin Islands” OR “Italy” OR “Slovak Republic” OR “Brunei” OR “Japan” OR “Slovenia” OR “Canada” OR “Korea” OR “Spain” OR “Cayman Islands” OR “Kuwait” OR “St. Kitts” OR “Nevis Channel Islands” OR “Latvia” OR “St. Martin” OR “Chile” OR “Liechtenstein” OR “Sweden” OR “Croatia” OR “Lithuania” OR “Switzerland” OR “Curacao’ OR “Luxembourg” OR “Taiwan” OR “Cyprus” OR “Macao” OR “Trinidad and Tobago” OR “Czech Republic” OR “Malta” OR “Turks and Caicos Islands” OR “Denmark” OR “Monaco” OR “United Arab Emirates” OR “Estonia” OR “Nauru” OR “United Kingdom” OR “Faroe Islands” OR “Netherlands” OR “United States” OR “Finland” OR “New Caledonia” OR “Uruguay” OR “France” OR “New Zealand” OR “Virgin Islands (U.S.)” OR “French Polynesia” OR “Northern Mariana Islands” OR “Germany” OR “Norway”OR “High income countr*” OR “upper-middle income countr*” OR “developed countr*” OR “developed nation*” OR “developed population*”

#### Inclusion criteria

We included all studies that involved the community, service users, consumers, households, patients, public and their representatives in the planning, implementation, monitoring and evaluation of health services, policy, or interventions. These included studies that involved the community in disease prevention, promotion, or healthy living, and/or health service delivery. Studies that involved patients in decision making of personal healthcare decisions only were excluded from our review. We also excluded studies where Community Based Participatory Research (CBPR) was used merely to suggest ideas rather than as part of implementation in a community program. For this review, we excluded editorials and theoretical studies but included reports which had a description of the community participation component. We did not impose any language restrictions but limited the search to published literature from high and upper-middle income countries as defined by the World Bank.

#### Search and retrieval of studies

**Fig 1:**
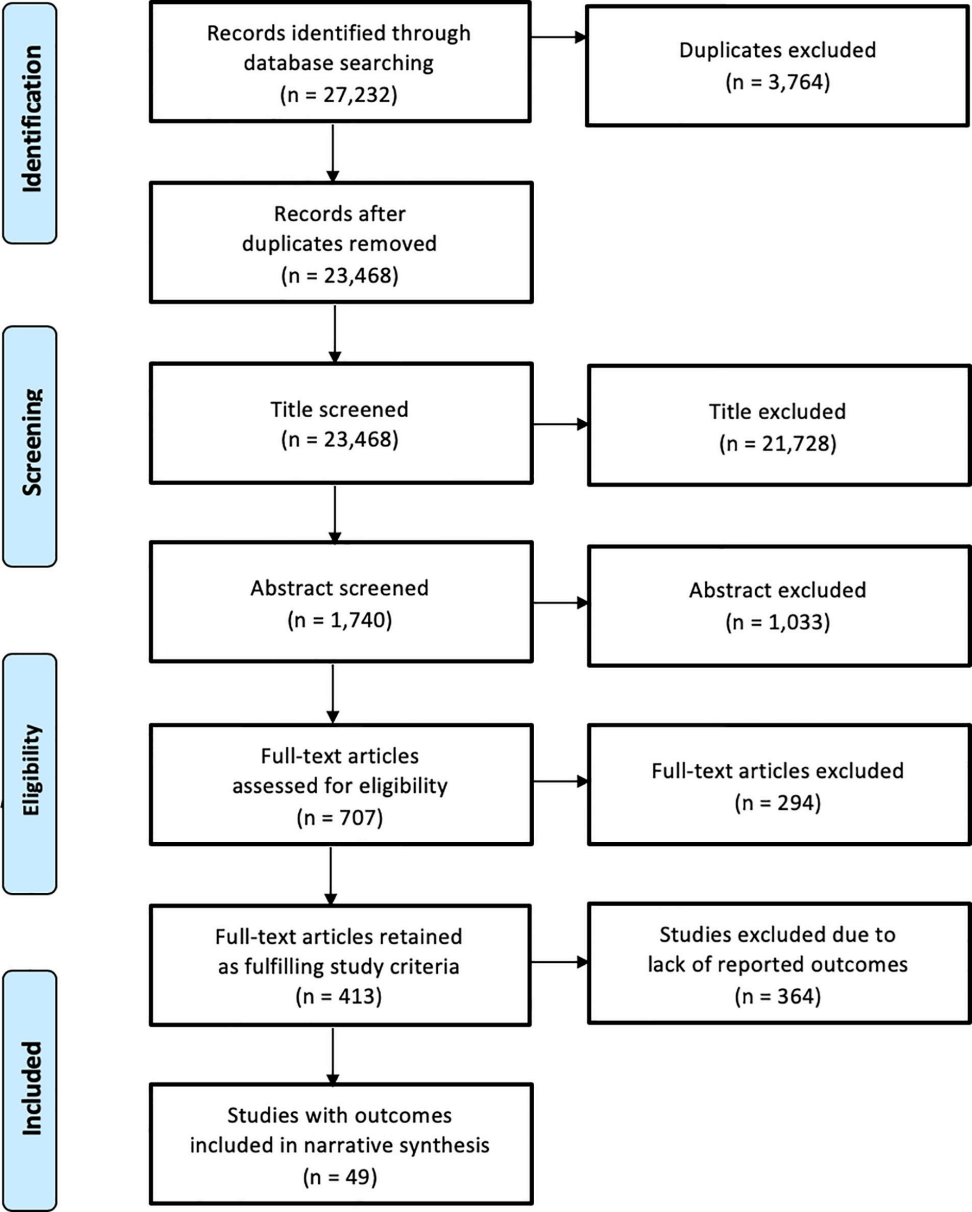
PRISMA flowchart.

Two reviewers (SS and AS) double screened titles and keywords for 20% of the total articles from the search in the databases (kappa coefficient = 0.82). The remaining 80% of the articles were distributed among SS and AS and screened only once due to the high initial Kappa coefficient. Following the title screenings, the abstracts included were double screened (kappa coefficient = 0.84). Any disagreement at this stage was discussed between SS and AS. In the absence of a consensus, opinion was sought from a third reviewer for resolution. Five reviewers (SS, AS, VH, FC, HLQ) conducted the full-text screening. Articles in languages other than English (e.g. French, German, Spanish, and Portuguese) were screened by a reviewer who could read and understand the article. Disagreements were resolved by a third reviewer. Only papers that reported outcomes or effects of community participation were included in this review. The details of the studies screened and included at each stage are presented in a flowchart in Fig 1.

#### Data synthesis

Two reviewers (VH and FC) conducted data extraction using standardized forms including categories on: (1) study characteristics including study design and setting, (2) type of community involvement described in the paper, and (3) outcomes reported. The two reviewers (VH and FC) met regularly to discuss and resolve any discrepancies or disagreements on the data extraction or interpretation of the studies. We conducted a narrative synthesis of the findings.

#### Risk of bias assessment

Two reviewers (VH and FC) assessed the studies for risk of bias. The Cochrane risk of bias tool was used to assess randomized control trials (RCTs) while observational studies were assessed using a proforma with 3 domains: selection bias, information bias, and confounding, then categorised as low, high, or unclear. Qualitative studies were evaluated for quality with an adapted checklist used in a previous series of mixed methods systematic reviews [13, 14] scored for ten core criteria. We classified studies with a score of eight to ten as having an overall low risk of bias, four to seven as having an overall medium risk of bias, and zero to three as having an overall high risk of bias. We did not conduct a risk of bias assessment on case studies; however, we have included these studies in our review as they give insight into the mechanisms of partnerships, inter-organisation collaboration, and stakeholder satisfaction.

## Results

27,232 records were identified through database searching. 23,468 articles were screened by title followed by 1,740 abstracts screened for inclusion. The full text of 707 articles was obtained and assessed for eligibility. After screening for reported objectives, 49 articles met eligibility criteria for this review (Fig 1). Due to the heterogeneity in study design, intervention types, participants, and outcomes, we conducted a narrative synthesis of the findings instead of a meta-analysis.

### Characteristics of included studies

Of the 49 studies that met inclusion criteria, 22 were quantitative, 14 were qualitative, and 13 were case studies. Of the 22 quantitative studies, 6 were RCTs, 8 were intervention studies, 7 were cohort studies, and 1 was a cross-sectional study. The studies could be categorised into five different disease categories based on the focus of the community participation initiative described. Of the 49 studies, 16 focused on community health in general, 13 involved initiatives that targeted healthy living, 9 focused on non-communicable diseases, 7 studies addressed infectious diseases, and 4 studies were related to environmental health. The description of each disease category and the number of relevant studies are presented in Table 2.

**Table 2:** Categories of community involvement initiatives (n = 49).

Category	Description	n
Community Health	Context specific and priority setting related initiatives for a range of health issues addressed at the community level.	16
Healthy Living	Initiatives focused on nutrition, physical activity and obesity.	13
Non-Communicable Diseases	Initiatives addressing conditions such as asthma, mental health, diabetes, substance abuse, etc.	9
Infectious Diseases	Initiatives addressing diseases such as HIV/AIDS, tuberculosis, parasitic diseases, dengue etc.	7
Environmental Health	Initiatives focused on environmental health or natural disaster responses.	4

**Fig 2:**
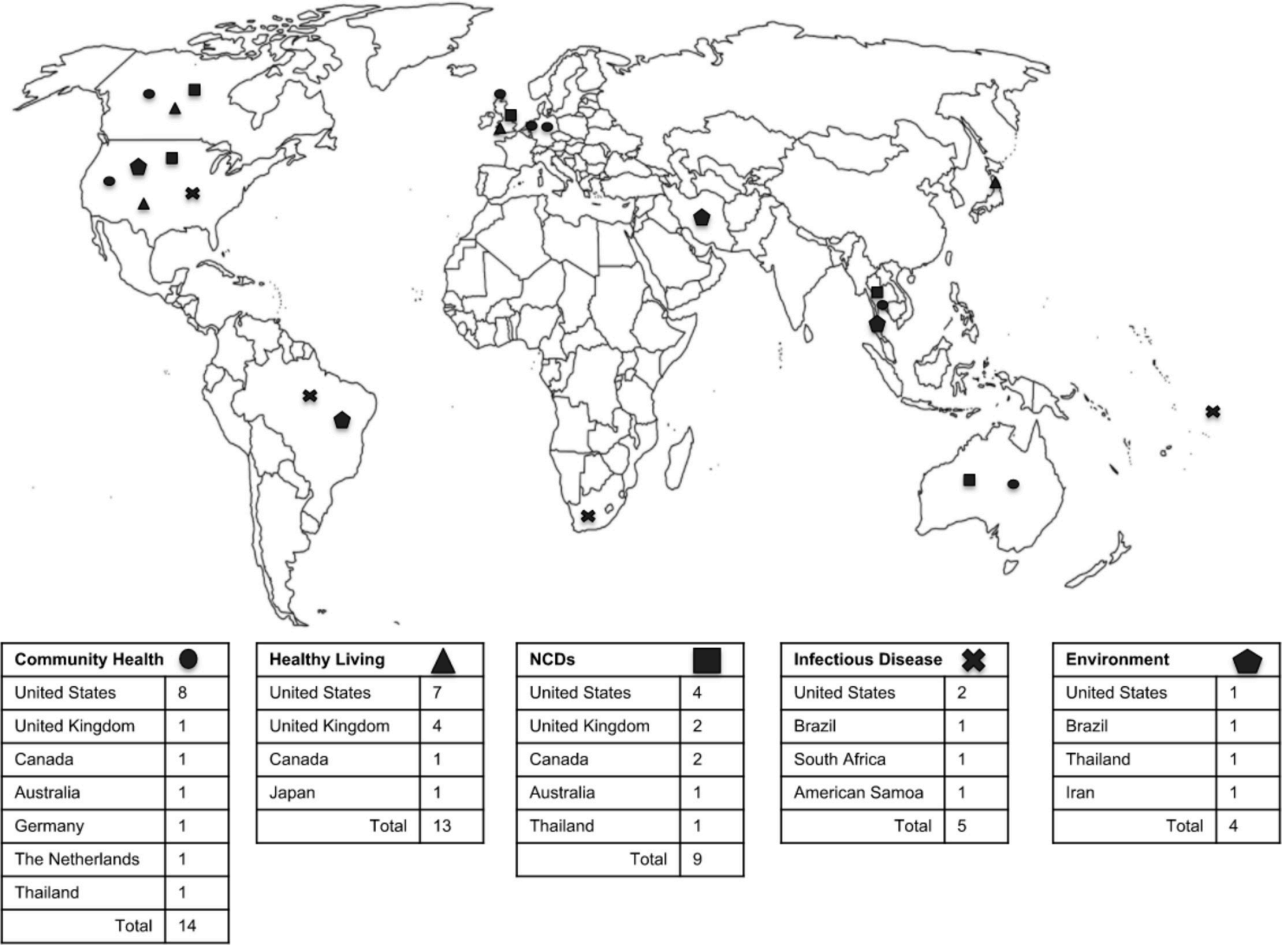
Study location by category.

Overall, studies were located in North America (n = 25), Europe (n = 9), Asia (n = 5), South America (n = 6), Africa (n = 1), and Oceania (n = 3) (Fig 2). The community health category featured the most geographic diversity with studies from nine different nations represented. The United States was represented by studies in all categories.

### Outcome definitions and framework

Reported outcomes were classified as process outcomes, community outcomes, health outcomes, stakeholder perspectives, and empowerment (See Table 3). We define process outcomes as short-term outputs that reflect the effectiveness of collaborative processes and activities over time. Organizational processes are concerned with community-based group achievements, while community processes are linked to process-related changes in the targeted community. We define community outcomes as intermediate social effects that represent changes in community member’s knowledge, attitudes, and behaviors. More extensively, it includes outcomes that reflect impact on social capital, community development, socio-cultural, and environmental improvements. Health outcomes are those that reflect changes in community member’s health status. We also describe those outcomes that deal with larger sociopolitical influences, as well as stakeholder perceptions. Studies also report on empowerment at the community or individual level, as an outcome. Studies that defined empowerment framed it as communities coming together to address a self-identified community problem and create positive change that is self-sustained, contextually appropriate, and fosters knowledge transfer between community members. These studies also point to complicated power relations and structural differences between community members and professionals or policy makers that underpin the challenges in defining and measuring community or individual empowerment (See Table 4).

**Table 3:** Outcomes definitions.

	Process Outcomes					
	Organisational Processes	Community Processes	Community Outcomes	Health Outcomes	Perspectives	Empowerment
**Definition**	Concerned with the formation, functioning and achievements of a community-based group or coalition	Linked to process-related changes identified in the targeted community such as increased community participation, outreach or uptake of services	Changes in the knowledge, attitudes and behaviours of members in the community on a targeted health issue	Changes in the health status of members of the community of concern	Stakeholder satisfaction or views with the processes of community involvement or with the outputs from those processes	Communities coming together to address a self-identified community problem and create positive change that is self-sustained, contextually appropriate and fosters knowledge transfer between community members
**Example**	*A coalition forms and through the process of developing and implementing a project*, *establishes new or better working relationships with other community organisations*	*A community-academic partnership holds a health fair where 150 people receive health education*, *20 people sign up to volunteer with the partnership*	*After an intervention on healthy living in a local park*, *surveyed community members report a greater awareness of the importance of physical activity and it can be seen by coalition members that the park is used more for jogging and fitness*	*A healthy living intervention leads to decreased BMI and waist circumference pre-post assessment*	*Members of a community academic coalition report that they enjoyed the process of working together and feel that they have created a worthwhile and useful program*	*Members of a community identify the need for dengue control and work together and with local NGOs to implement dengue prevention measures and community groups provide dengue education at churches and schools*

**Table 4:** Definitions of empowerment reported in studies included.

Definition of Empowerment	Category	Author/Date
“Individual levels of empowerment" described in terms of youth's ability to "reach out" and disseminate health information to the community. Focus on reaching out to and advocating for undocumented immigrants and helping them to gain confidence, knowledge and access services while "feeling empowered to motivate others to do the same."	Community Health	Ferrera et al 2015 [15]
"When local people at all levels are drawn together with the purpose of employing local wisdom to solve a problem which they all face, the result is a sense of empowerment to make changes, which are intrinsically sensitive to local circumstances, widely accepted by the community, and because of this, more likely to be sustained"	Environmental Health	Sansiritaweesook et al 2015 [16]
"Empowerment is related to the process of giving groups of communities autonomy and a progressive and self-sustained improvement of their lives."	Infectious Disease	Caprara et al 2015 [17]

**Fig 3:**
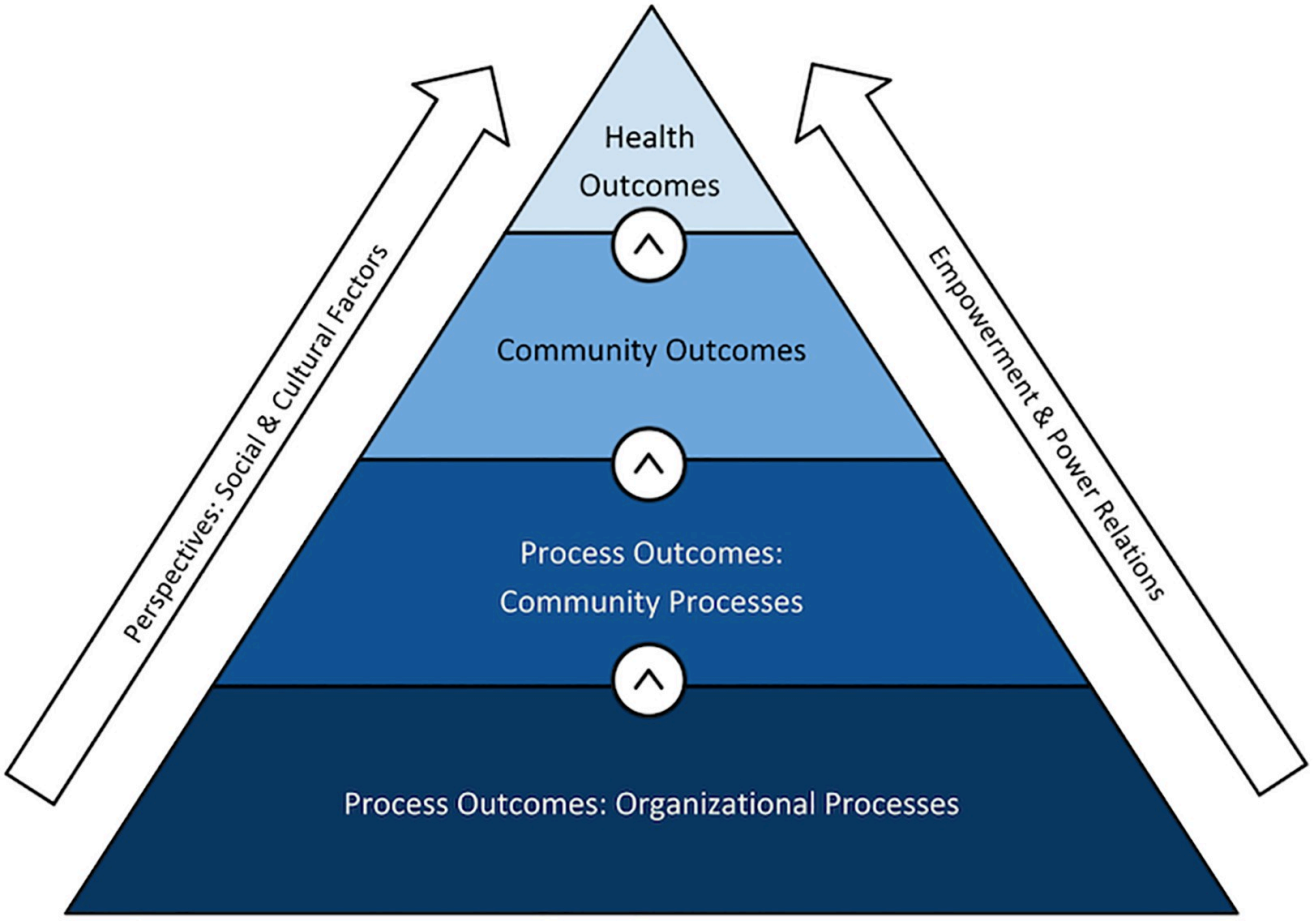
Community participation outcomes framework.

Outcomes of community involvement initiatives may be viewed through a hierarchy, as some outcomes necessitate others (See Fig 3); for example in order to deliver a community involvement program that reports robust health outcomes, it is important to have functional and sustainable underlying organisational structures, as well as community awareness and involvement. Throughout this hierarchy, both organisation and community members may report perspectives on the process or outputs and may feel empowered at either a personal or community level.

The number of outcomes reported by disease category and study design can be found in Table 5. Twenty-nine studies reported process outcomes, of which twenty-three reported organisational processes and nine reported community processes; twenty-one studies reported community outcomes; sixteen reported perspectives of stakeholders on either processes or project outcomes; six reported on empowerment and twelve reported health outcomes. Process outcomes, especially organisational processes, were most often reported in studies involving community health (n = 12), while both infectious disease and environmental health category only had one study reporting these outcomes. Empowerment was the least reported across study categories; of 6 studies, 4 were in the community health category. Health outcomes were more often reported in healthy living (n = 4) and non-communicable disease initiatives (n = 5), while community health initiatives reported no health outcomes.

**Table 5:** Outcomes by study design and disease category.

Disease Category	Study Design	Outcomes (n = )					
		Process Outcomes—Organizational Processes	Process Outcomes—Community Processes	Community Outcomes	Stakeholder Perspectives	Empowerment	Health Outcomes
Community Health	RCT (n = 1)	1	0	0	0	0	0
	Intervention study (n = 1)	0	0	0	1	0	0
	Cohort (n = 3)	1	1	0	1	0	0
	Qualitative (n = 7)	6	1	2	4	3	0
	Case Study (n = 4)	4	1	1	0	1	0
	**∑**	**12**	**3**	**3**	**6**	**4**	**0**
Healthy Living	RCT (n = 2)	0	0	2	0	0	1
	Intervention study (n = 3)	1	0	1	1	0	2
	Cohort (n = 1)	0	0	1	0	0	1
	Cross-sectional study (n = 1)	1	0	0	0	0	0
	Qualitative(n = 3)	1	0	1	2	1	0
	Case Study (n = 3)	2	1	1	1	0	0
	**∑**	**5**	**1**	**6**	**4**	**1**	**4**
Non Communicable Diseases	RCT (n = 1)	1	0	0	0	0	0
	Intervention study (n = 2)	1	0	1	0	0	2
	Cohort (n = 3)	2	0	0	0	0	2
	Qualitative (n = 1)	0	1	0	0	0	0
	Case Study (n = 2)	0	1	1	1	0	1
	**∑**	**4**	**2**	**2**	**1**	**0**	**5**
Infectious Diseases	RCT (n = 1)	0	1	1	1	0	1
	Intervention study (n = 1)	0	1	1	0	0	0
	Qualitative (n = 2)	1	0	2	0	0	0
	Case Study (n = 3)	0	1	3	2	0	1
	**∑**	**1**	**3**	**7**	**3**	**0**	**2**
Environmental Health	RCT (n = 1)	0	0	1	0	0	0
	Intervention study (n = 1)	1	0	1	1	0	1
	Qualitative(n = 1)	0	0	0	1	0	0
	Case Study (n = 1)	0	0	1	0	1	0
	**∑**	**1**	**0**	**3**	**2**	**1**	**1**
**∑**		**23**	**9**	**21**	**16**	**6**	**12**

### Process outcomes

Study characteristics, along with the findings reported, and the risk of bias assessments for studies that report on process outcomes can be found in Table 6 (See S1 File for table legend for risk of bias).

**Table 6:** Study characteristics, findings reported and the risk of bias assessments for studies that report on process outcomes (n = 28).

**Study**	**Country**	**Study Design**	**Sample**	**Disease Category**	**Type of** **Community Involvement**	**Type of Outcome**	**Relevant Findings**							**Risk of bias**					
														**s**	**p**	**d**	**a**	**r**	**Overall**
**Gloppen et al 2012 [18]**	United States	RCT	12 pairs of matched communities in 7 states	Community Health	Communities That Care (CTC) coalitions—mobilize stakeholders to implement prevention programs to promote adolescent health and wellbeing.	Process Outcome	20 months after study support ended which included tailored training, technical assistance, and funding: 1) 11 of the 12 CTC coalitions still existed. 2) CTC coalitions maintained a relatively high level of implementation fidelity to the CTC system.												Medium
**Boivin et al** **2014 [19]**	Canada	RCT	172 individuals from 6 communities	Non Communicable Disease	Communities involved to set priorities for improving chronic disease management in primary care.	Process Outcome	1) Priorities established with patients were more aligned with components of the Medical Home and Chronic Care Model (p < 0.01). 2) Priorities established by professionals alone placed more emphasis on technical quality of disease management. 3) 41% increase in agreement on common priorities (95%CI: +12% to +58%, p < 0.01). 4) Patient involvement increased the costs of the prioritization process by 17%, and required 10% more time to reach consensus on common priorities.												Low
**Study**	**Country**	**Study Design**	**Sample**	**Disease Category**	**Type of** **Community Involvement**	**Type of Outcome**	**Relevant Findings**							**Risk of bias**					
														**s**	**p**	**d**	**a**	**r**	**Overall**
**Sansiritaweesook et al 2015 [16]**	Thailand	Intervention study	182 informants, 562 surveillance networks, 21,234 villagers	Environmental Health	7-step process used to develop a model for local drowning surveillance system based on community participation.	Process Outcome	1) Villagers collaborated to conduct a situation analysis, design, and trial a prototype intervention, scale up to a full system design and trial that was followed by system improvement and dissemination. 2) 80% of networks were cooperative in submitting timely reports and using them for action. 3) Accuracy of information in reports increased from 65% to 90%.												Medium
**Hoelscher et al 2010 [20]**	United States	Intervention study	15 schools receive BPC intervention, matched with 15 schools that receive BP only	Healthy Living	School-based obesity prevention program (CATCH BP) versus complimentary program (CATCH BPC) that formed partnerships with external community organizations.	Process Outcome	1) BPC schools demonstrated better outcomes with more activities and lessons than BP schools. 2) In year 2 there was a higher mean number of physical activity and healthy eating programs being implemented in BPC schools (mean = 3.71 programs) compared to BP schools (mean = 2.73 programs).												Unclear
**Neto et al 2003 [21]**	Brazil	Intervention Study	1,524 households in intervention area; 1,564 households in control area	Infectious Disease	A preliminary diagnosis presented to the community to launch a discussion aimed at defining future actions, implementation of the actions in the study area with community participation.	Process Outcome	Changes in the study area included: vector control workers began demonstrating preventive measures without removing potential breeding places or using larvicide; use of educational aids specific to the local reality; activities related to the residents’ priorities; and activities such as music, theater skits, scavenger hunts, and games to demonstrate the vector cycle.												Unclear
**Clark et al** **2014 [22]**	United States	Intervention study	1,477 parents of children with asthma in coalition target areas and comparison areas	Non Communicable Disease	Allies Against Asthma program—a 5-year collaborative effort by 7 community coalitions designed to change policies regarding asthma management in low-income communities of color.	Process Outcome	89 inter- and intra-institutional changes were made on systems and policies to statewide legislation across the 7 communities.												Unclear
**Study**	**Country**	**Study Design**	**Sample**	**Disease Category**	**Type of** **Community Involvement**	**Type of Outcome**	**Relevant Findings**							**Risk of bias**					
														**s**	**d**	**n**	**c**	**Overall**	
**Nathan et al** **2006 [23]**	Australia	Cohort	47 staff in 2001; 43 in 2002	Community Health	Community Representatives Program—community members provided the opportunity to give input on service delivery issues and needs in the community and to be active participants in committee work of the health service through participation in decision-making committees with other stakeholders.	Process Outcome	1) Significantly more staff at the follow-up survey reported that they and other staff were clear about the role of community representatives and how to work with them on committees. 2) Significantly more staff at follow-up felt that the health service was ready for this type of initiative.											Unclear	
**Akiyama et al 2013 [24]**	Thailand	Cohort	43 primary-level schools	Community Health	Health Promoting School program with the aim of encouraging schools to improve school health. Interventions include 6 one-day training workshops and an action plan support involving teachers.	Process Outcome	1) Increase in school and community partnership [mean score 1.0 pre (median = 1.0, IQR = 0.5–1.5) vs. 2.4 post (median = 2.5, IQR = 2.0–3.0)]. 2) Improvements in the definition of the roles and responsibilities with the Burmese community [mean score 0.4 pre (median = 0, IQR = 0) vs. 2.7 post (median = 3.0, IQR = 3.0–3.0)].											Medium	
**Reeve et al** **2015 [25]**	Australia	Cohort	N/A	Non Communicable Diseases	A health service partnership between an Aboriginal community-controlled health service, a hospital, and a community health service that implemented an integration of health promotion, health assessments, and chronic disease management.	Process Outcome	Short-term outcomes– 1) Increase in occasions of service (from 21,218 to 33,753) particularly in PHC in remote areas (from 863 to 11,338). 2) Increased uptake of health assessment (from 13% of eligible population to 61%, then to 73% of those identifies with DM placed on a care plan). Medium-term outcomes– 1) Over a 6 year period, improvements in quality-of-care indicators, i.e. glycated hemoglobin checks and proportion of people with DM receiving anti hypertensives. 2) Increase in proportion of patients identified with chronic disease or risk factors. 3) Increased PHC episodes and follow-up.											Medium	
**Nelson et al** **2006 [26]**	Canada	Cohort	79 Consumer Survivor Initiative members	Non Communicable Diseases	Consumer Survivor Initiatives—organizations that are operated by and for people with a history of mental illness.	Process Outcome	Members participated most often in internal activities (e.g. social-recreational, committees) and least often in external activities (e.g. advocacy, planning, education) with an average of 3 activities per month.											Low	
**Litt et al** **2013 [27]**	United States	Cross-sectional	59 collaborative groups representing 22 states	Healthy Living	Collaboratives formed to improve the built environment and policies for active living.	Process Outcome	Groups made progress in identifying areas for environmental improvements and in many instances received funding to support these changes: 1) Groups’ environmental improvement scores ranged from 1.5 to 5.0, with an average of 3.5 (SD: 0.9). This average indicated that groups typically had funding to support their initiatives and had started but had not completed the planned improvements. 2) Groups’ policy change scores ranged from 1.0 to 4.5 with an average of 2.9 (SD: 1.0), suggesting that groups had generally identified a policy gap and had started discussions to develop new policies or changes to existing policy.											High	
**Study**	**Country**	**Study Design**	**Sample**	**Disease Category**	**Type of** **Community Involvement**	**Type of Outcome**	**Relevant Findings**	**Risk of bias**											
								**1**	**2**	**3**	**4**	**5**	**6**	**7**	**8**	**9**		**Overall**	
**Gibbons et al 2016 [28]**	United States	Qualitative	3 focus groups, 8 in-depth interviews, 31 individuals surveyed	Community Health	Community-academic collaboration using CBPR known as the 'Community Health Initiative: Creating a Healthier East Baltimore Together.'	Process Outcome	1) Enabled the development of authentic community-academic relationships. 2) Enabled establishment of a “level playing field” among residents. 3) Enabled change in attitudes, perceptions among personnel and residents of each other. 4) Enabled residents to become active participants of the decision-making process.	N	Y	Y	Y	Y	N	Y	N	N	N	Medium (5/10)	
**Trettin et al** **2000 [29]**	United States	Qualitative	6 to 14 participants of 3 focus groups (total n = 60)	Community Health	Volunteer-based community health advisory program developed to increase residents' access to health services, stimulate their interest in health, disease prevention, and awareness of health-related environmental issues, and empower residents to be more involved in community health.	Process Outcome	1) Planning approach for the program identified as appropriate for local context. 2) Existing list of problems and needs identified as accurate with perspectives of local participants. 3) Field-workers established good relationships with the community.	N	Y	Y	Y	Y	Y	Y	N	N	Y	Medium (7/10)	
**Carlisle et al** **2010 [30]**	United Kingdom	Qualitative	Not mentioned. Semi-structured interviews	Community Health	'Social Inclusion Partnerships'—organized around committee-style management board meetings attended by members from statutory, voluntary, and community sectors.	Process Outcome	Drug and alcohol misuse classified as a particular problem amongst younger people.	Y	N	Y	N	Y	N	N	N	N	N	High (3/10)	
**Johnson et al 2006 [31]**	United States	Qualitative	40 community based organizations (CBOs) selected for interview	Community Health	CBOs involved in implementing health-related projects through locally administered micro-grants. 'The Healthy Carolinians Community' serving as grantors partnering with the CBOs	Process Outcome	Microfinancing CBOs aided in: 1) Building partnerships and connections within and outside their communities. 2) Gained new ideas and knowledge. 3) Developed local leadership and expertise. 4) Increased their ability to focus on and progress towards goals.	N	Y	Y	Y	Y	Y	Y	N	N	Y	Medium (7/10)	
**Ferrera et al 2014 [15]**	United States	Qualitative	23 youths interviewed	Community Health	CBPR used to form a Youth advisory board. Youth involved in decision making and programming, as well as in a feedback and improvement role.	Process Outcome	1) Students feel consistently comfortable with program staff and the sense that a personal and emotional investment was made. 2) Program participants went on to give health education to approximately 800 community members. 3) 500 community members attended the health fair hosted by a participating school.	Y	Y	Y	Y	Y	N	N	N	N	Y	Medium (6/10)	
**Heaton et al 2014 [32]**	United States	Qualitative	Interviews, focus groups	Community Health	Collaborative partnership between 2 academic health centers and CBOs to determine topics, and develop a bi-directional educational seminar series called 'Community Grand Rounds' (CGR).	Process Outcome	1) Partnership had good adherence to principles of collaborative and equitable group process in planning of the CGR event. 2) Educational seminars facilitated bi-directional communication between their community and university medical center. 3) Format and content of seminars effectively tailored to unique needs of each community.	N	Y	Y	Y	Y	Y	Y	Y	N	Y	Low (8/10)	
**Litt et al 2013 [33]**	United States	Qualitative	59 participants from collaboratives interviewed	Healthy Living	Multi-sectoral collaborative groups promote active lifestyles through environmental and policy changes.	Process Outcomes	1) Groups reported working on an average of 5 strategy areas including parks and recreation (86%), Safe Routes to School (85%), street improvements (78%) and streetscaping (69%). 2) More than half of groups reported their environmental initiatives as either in progress or completed. 3) Groups reported the most success in changing policy for public plazas, street improvements, streetscaping, and parks, open space, and recreation.	N	Y	N	N	N	N	N	N	N	N	High (1/10)	
**Rutter et al 2004 [34]**	United Kingdom	Qualitative	32 interviews conducted in one trust and 17 interviews in another trust of service users and sector reps	Non Communicable Diseases	User or patient involvement in the planning and delivery of health services through meetings between service managers and users; development of documents by user groups; service provider (Trust) meetings involving user representation.	Process Outcomes	Positive outcomes of user involvement reflected in their participation in campaigns against Trust plans; in refurbishing of inpatient units; in contract specification and monitoring of hotel services; in policy, practice and information about women's safety; and in integration of health and social services.	N	Y	Y	Y	Y	N	Y	N	N	N	Medium (5/10)	
**Chervin et al 2005 [35]**	United States	Qualitative	364 in-person interviews with project staff, evaluators, and community and agency members	Infectious Diseases	Center for Disease Control and Prevention’s Community Coalition Partnership Program–building a community’s capacity to prevent teen pregnancy through strengthening of partnerships, mobilization of community resources, and changes in the number and quality of community programs.	Process Outcome	1) Partners worked together to reduce duplication and fill gaps in services through collaboration and differentiation of activities. 2) Development of new programs from the partnership. It was noted however that increased partner skill, program improvements, and new programs did not appear sufficient to affect community capacity.	N	Y	Y	Y	Y	Y	Y	N	N	N	Medium (6/10)	
**Study**	**Country**	**Study Design**	**Sample**	**Disease Category**	**Type of** **Community Involvement**	**Type of Outcome**	**Relevant Findings**											**Risk of Bias**	
**von dem Knesebeck et al 2002 [36]**	Germany	Case Study	Not mentioned	Community Health	A community-level health policy intervention: 'Local Co-ordination of Health and Social Care' project.	Process Outcome	1) 70% agreement between managers of Project Offices, moderators and other key actors on usefulness of 'Round Table' to improve coordination of health and social care at the community level. 2) Success in the development and enactment of recommendations for action programs e.g. improved information dissemination, further development of geriatric ambulatory rehabilitation. 3) Development of health monitoring and reporting activities at the community level. 4) Improved cooperation among participating communities through increased transparency.											N/A	
**Keene Woods et al 2014 [37]**	United States	Case Study	Not mentioned	Community Health	Community Change Intervention that focused on building coalition capacity to support implementation of community changes for program, policy, and practice.	Process Outcome	1) Coalition facilitated an average of at least 3 times as many community changes (i.e., program, policy, and practice changes) per month following the intervention. 2) After intervention, there was increased implementation of 3 key prioritized coalition processes: Documenting progress/using feedback (75% increase in stakeholders involved in designing the documentation system); making outcomes matter (50 to 100% increase in activities relating to incentives, accountability, and use of longer term outcomes with accountability); and sustaining the work (42% to 75% increase in identification of sustainability decision makers, determining what to sustain and duration of sustained effort). 2) A 1-year probe following the study showed that majority of the community changes were sustained.											N/A	
**Bursztyn et al 2008 [38]**	Brazil	Case Study	Not mentioned	Community Health	A project was developed and implemented in primary health centers to improve young men’s adherence to a teenage health care program using participatory planning techniques, and rapid assessment procedures.	Process Outcome	1) Self-assessment workshops were held with the local teams. Despite good awareness among the health professionals, the project’s results varied between health centers. Over-centralization and lack of flexibility appear to be related to lower capacity to incorporate new practices. 2) Health centers where specific strategies were observed showed more successful results.											N/A	
**Orozco-Núñez et al 2009 [39]**	Mexico	Case Study	Not mentioned	Community Health	Use of participative strategies and the creation of support networks for poor pregnant women.	Process Outcome	Coordination and community participation were relevant in relation to major resources allocation and availability, particularly housing and transportation.											N/A	
**Baker et al** **2012 [40]**	United States	Case Study	Not mentioned	Healthy Living	'Active Living by Design' partnerships were established to change environments and policies, and support complementary programs and promotions to increase physical activity.	Process Outcome	The connections among diverse community partners created a foundation that enhanced lead agency efforts to form, implement, and maintain policy changes and physical projects, as well as promotional and programmatic approaches, to support active living.											N/A	
**Rapport et al 2008 [41]**	United Kingdom	Case Study	Focus groups with project steering group	Healthy Living	Action research project–organized to respond to a context of funding and service delivery, helmed by a Project Steering Group made up of community members, study organizers, statutory board members.	Process Outcome	1) Community members involved acquired new skills and "strengthened individual competencies," heightened knowledge amongst the community and Project Steering Group of community members' needs and desires," influenced working practices, altered perspectives and raised awareness of issues surrounding trust and communication within partnerships. 2) The data generated by the community interviews was perceived as more robust evidence that could be "taken seriously and gave credibility to the communities' comments and requests."											N/A	
**Diaz et al 2009 [42]**	Cuba	Case Study	Not mentioned	Infectious Diseases	Ecohealth approach used as a strategy to ensure active participation by the community, diverse sectors, and government. The approach allowed holistic problem analysis, priority setting, and administration of solutions.	Process Outcome	1) The strategy had been sustained two years after concluding the process. 2) 93.5% had attended trainings under the project and 89% knew that the inhabitants of the neighborhood had organized themselves into groups promoted by the project. 3) 93.5% considered that the community improved its ability to identify problems that affected its ecosystem and proposed solutions.											N/A	
**Barnes et al** **2006 [43]**	United Kingdom	Case Study	Not mentioned	Non Communicable Diseases	Users of a community mental health inter-professional training program (partnerships with service users) involved in the commissioning, management, delivery, participation, and evaluation of the program, as trainers and as course members.	Process Outcome	Commitment to partnership established, reinforced by service users participating in the commissioning of the program and its evaluation, e.g. service users took active part in the steering group that advised research.											N/A	

Nine studies presented process outcomes relating to contextually appropriate initiatives and mutually agreeable organizational processes to meet community’s needs [15, 16, 25, 26, 28–30, 44, 45]. Four studies reported on how collaborative processes led to the creation of appropriate policies and community-led priority setting [19, 22, 34, 43]. Two studies reported clearer role definition as a process outcome of community involvement in community health initiatives [3, 46] while two studies reported how robust processes enabled the provision of more activities [20, 47]. Yet, not all partnerships showed favorable results, due to conflicting stakeholder views, as well as underestimation of the time and resources required for collaboration [35].

### Community outcomes

Study characteristics, along with the findings reported and the risk of bias assessments for studies that report on community outcomes can be found in Table 7 (See S1 File for table legend for risk of bias).

**Table 7:** Study characteristics, findings reported and the risk of bias assessments for studies that report on community outcomes (n = 20).

**Study**	**Country**	**Study Design**	**Sample**	**Disease Category**	**Type of** **Community Involvement**	**Type of Outcome**	**Relevant Findings**							**Risk of bias**					
														s	p	d	a	r	Overall
**Ardalan et al** **2010 [48]**	Iran	RCT	15 intervention villages and 16 control villages	Environmental Health	Intervention assembles Village Disaster Taskforces (VDTs), conducts training of VDTs and community, evacuation drills, and program monitoring.	Community Outcome	1) Adjusted odds ratio for participation in an evacuation drill in intervention area post vs. pre-assessment was 29.05 (CI: 21.77–38.76) compared to control area 2.69 (CI: 1.96–3.70) (p<0.001). 2) Participation in a family preparedness meeting and risk mapping were helpful in motivating individuals to take preparedness actions.												Medium
**Solomon et al 2014 [49]**	United Kingdom	RCT (Stepped wedge cluster)	10,412 adults (intervention = 4693; control = 5719)	Healthy Living	Intervention developed with local partners using local knowledge and resources to facilitate local involvement in planning, promotion, and delivery of a physical activity intervention.	Community Outcome	Low penetration of intervention wherein 16% of intervention participants reported awareness of intervention and 4% reported participating in intervention events.												High
**Derose et al** **2014 [50]**	United States	RCT	33 intervention parks (2 interventions, 17 control parks	Healthy Living	CBPR approaches used to increase park use and physical activity across 33 neighborhoods.	Community Outcome	Intervention parks invested in new and diversified signage, promotional items, outreach or support for group activities like fitness classes and walking clubs, and various marketing strategies; working with departmental management established structures for community input and park policy facilitated implementation and sustainability.												High
**Caprara et al** **2015 [17]**	Brazil	RCT	10 intervention clusters, 10 control clusters	Infectious Disease	Intervention adopted an Ecohealth approach to involve community through workshops, clean up campaigns, mobilization of school children and seniors, and distribution of information, education, and communication materials.	Community Outcome	Increase in peoples’ knowledge of dengue and willingness to participate in preventive actions.												Low
**Study**	**Country**	**Study Design**	**Sample**	**Disease Category**	**Type of** **Community Involvement**	**Type of Outcome**	**Relevant Findings**							**Risk of bias**					
														s	p	d	a	r	Overall
**Sansiritaweesook et al 2015 [16]**	Thailand	Intervention study	182 informants, 562 surveillance networks, 21,234 villagers	Environmental Health	7-step process used to develop a model for local drowning surveillance system based on community participation.	Community Outcome	Additional drowning prevention and rescue devices made available at high risk water resources. Proportion of sites with devices increased from 18.4% to 83.7%. Sites with security measures increased from 13.2% to 76.7%. Level of surveillance at high risk sites rose from 88.4% to 100%. Children 7–15 years who could swim rose from 38.5% to 52% following swimming lessons. Training of rescue volunteers in CPR increased from 6% to 27.4%. Proportion of village health workers trained in CPR increased from 12.7% to 87.9%.												Medium
**Yajima et al** **2001 [51]**	Japan	Intervention study	20 participants each from 13 municipalities (intervention group), 2000 in reference group	Healthy Living	Health promotion program consisting of a community leaders committee trained to conduct health promotion activities.	Community Outcome	Intervention group pursued healthier lifestyles than the comparison group. 22% of the Intervention group and 4% of the comparison group frequently obtained information from health professionals. 29.8% of the intervention group and 10.8% of the comparison group were satisfied with their access to health-related information. Significantly more people in the Intervention group were doing exercise, eating meals regularly, paying attention to nutritional balance and to food additives, were interested in health, and were satisfied with access to health information after excluding the effects of age and socio-economic factors (p<0.05). People in the intervention group were significantly more likely to have greater health literacy regardless of socio-economic status.												Unclear
**Neto et al 2003 [21]**	Brazil	Intervention Study	1,524 households in intervention area; 1,564 households in control area	Infectious Disease	A preliminary diagnosis presented to the community to launch a discussion aimed at defining future actions, implementation of the actions in the study area with community participation.	Community Outcome	Potential domiciliary breeding sites were significantly reduced; the proportion of houses without breeding sites was significantly increased; and there was an increase in the percentage of individuals who recognized the larval form of the vector in the study area as compared to the control area.												Unclear
**Clark et al** **2014 [22]**	United States	Intervention study	1,477 parents of children with asthma in coalition target areas and comparison areas	Non Communicable Disease	Allies Against Asthma program—a 5-year collaborative effort by 7 community coalitions designed to change policies regarding asthma management in low-income communities of color.	Community Outcome	Allies parents, significantly more so than the comparison group parents, felt less helpless or frightened when confronted by a symptom episode (mean score change: 0.30 vs. 0.75; p = 0.014) and less angry about their child’s asthma (mean score change: 0.16 vs. 0.57; p = 0.011). Allies parents exhibited a greater increase in concern than did comparison parents about medications and side effects (mean score change: 1.22 vs. 0.79; p = 0.022), indicating higher awareness.												Unclear
**Study**	**Country**	**Study Design**	**Sample**	**Disease Category**	**Type of** **Community Involvement**	**Type of Outcome**	**Relevant Findings**							**Risk of bias**					
														s	d	n	c	Overall	
**Davison et al** **2013 [46]**	United States	Cohort	423 children age 2–5	Healthy Living	CBPR used to develop and pilot test a family-centered intervention for low-income families with preschool-aged children.	Community Outcome	Parents at post intervention reported significantly greater self-efficacy to promote healthy eating in children and increased support for children’s physical activity. Dose effects observed for most outcomes.											Low	
**Study**	**Country**	**Study Design**	**Sample**	**Disease Category**	**Type of** **Community Involvement**	**Type of Outcome**	**Relevant Findings**	**Risk of bias**											
								1	2	3	4	5	6	7	8	9		Overall	
**Ferrera et al** **2014 [15]**	United States	Qualitative	23 youths interviewed	Community Health	CBPR used to form Youth advisory board and youth involved in decision making and programming, as well as in a feedback and improvement role.	Community Outcome	Greater knowledge of health issues and the importance of screening.	Y	Y	Y	Y	Y	N	N	N	N	Y	Medium (6/10)	
**Heaton et al** **2014 [32]**	United States	Qualitative	Interviews, focus groups	Community Health	Collaborative partnership between 2 academic health centers and CBOs to determine topics, and develop a bi-directional educational seminar series called 'Community Grand Rounds'.	Community Outcome	Increased knowledge and awareness on health and social issues among community; Improved trust between academic partners, and community.	N	Y	Y	Y	Y	Y	Y	Y	N	Y	Low (8/10)	
**Litt et al** **2013 [33]**	United States	Qualitative	59 participants from collaboratives interviewed	Healthy Living	Multi-sectoral collaborative groups promote active lifestyles through environmental and policy changes	Community Outcomes	Most groups achieved some form of environmental or policy change.	N	Y	N	N	N	N	N	N	N	N	High (1/10)	
**Campbell et al 2001 [52]**	South Africa	Qualitative	30 members of community interviewed	Infectious Diseases	A community-based peer education program led by sex workers as an initiative in grassroots participation in sexual health promotion.	Community Outcomes	Increased confidence and personal development among peer educators and increased confidence among some sex workers.	Y	Y	Y	Y	N	N	Y	N	Y	Y	Medium (7/10)	
**Chervin et al** **2005 [35]**	United States	Qualitative	364 in-person interviews with project staff, evaluators, and community and agency members	Infectious Diseases	Centers for Disease Control and Prevention’s Community Coalition Partnership Program (CCPP)—building a community’s capacity to prevent teen pregnancy through strengthening of partnerships, mobilization of community resources, and changes in the number and quality of community programs.	Community Outcome	1. Increased community awareness of the problem of teen pregnancy and willingness to discuss the issue; 2. Improved knowledge and skills relating to addressing teen pregnancy.	N	Y	Y	Y	Y	Y	Y	N	N	N	Medium (6/10)	
**Study**	**Country**	**Study Design**	**Sample**	**Disease Category**	**Type of** **Community Involvement**	**Type of Outcome**	**Relevant Findings**											**Risk of Bias**	
**Orozco-Núñez et al 2009 [39]**	Mexico	Case Study	Not mentioned	Community Health	Use of participative strategies and the creation of support networks for poor pregnant women.	Community Outcome	Governmental actors’ involvement and leadership favored linking and coordination. Authorities, relatives, volunteers and users supported the referrals for obstetric emergencies, the identification of pregnant women in isolated areas, and their referral to health services. Around one-third of the users indicated geographical, economic, and cultural access barriers to health services in the four states, particularly those living in rural areas. Even though most of the informants received timely attention with a favorable evaluation of the treatment received in the units, testimonies were collected from users reporting feeling abused by transporters and suppliers.											N/A	
**Setti et al 2010 [53]**	Brazil	Case Study	24 participants	Environmental Health	The Neighborhood Ecological Program that involved the participation and empowerment of citizens in health promotion and sustainable development	Community Outcome	The program is reported to promote empowerment and community strengthening, dissemination of information and knowledge, development of critical thinking, and the creation of support networks.											N/A	
**Barnes et al** **2006 [43]**	United Kingdom	Case Study	Not mentioned	Non Communicable Diseases	Users of a community mental health inter-professional training program (partnerships with service users) involved in the commissioning, management, delivery, participation, and evaluation of the program, as trainers and as course members.	Community Outcome	1) Increase in mean of 'knowledge of factors involved in facilitating therapeutic cooperation' [5.8 (2.2 SD) vs. 8.3 (1.2 SD), p<0.001]. 2) Increase in mean of 'skills in facilitating therapeutic cooperation' [5.9 (2.3 SD) vs. 8.2 (1.3 SD), p<0.001]. 3) Increased in mean of 'A user-and carer- oriented perspective based on partnership in the provision of assessment, treatment and continuing care' [6.0 (2.1 SD vs. 8.5 (1.2 SD), p<0.001)]. 4) Increased knowledge on learning where and how to access information, developing directories of local service user groups/resources, and understanding the value of advocacy. 5) Positive changes in attitudes towards partnership with service users. 6) Positive changes in behavior at individual level, e.g. students more conscious of sharing decision-making and using a needs-led approach following awareness of the imbalance of power between service users and professionals. 7) Positive changes in behavior at organizational level, e.g. the setting up of service user groups, ensuring user views are fed into planning decisions, supporting service users on staff recruitment panels, writing leaflets for users/carers about services offered, and collating info on resources for users.											N/A	
**Wilson et al** **2014 [54]**	United States	Case Study	71 participants	Infectious Diseases	CBPR used to develop the Barbershop Talk With Brothers (BTWB) program—a community-based HIV prevention program that seeks to improve individual skills and motivation to decrease sexual risk, and that builds men’s interest in and capacity for improving their community’s health.	Community Outcome	1) Proportion of men who reported not having engaged in unprotected sex in past 3 months increased from baseline to follow-up administration of survey (25% to 41%, p = 0.007). 2) Proportion of men who reported having unprotected sex with two or more women in the past 3 months declined (46% to 17%, p = 0.0001). 3) Proportion of men reporting favorable attitudes towards condoms and confidence in their self-efficacy to use condoms consistently increased (p<0.05). 4) HIV stigma decreased, but difference did not reach statistical significance (Mean = 24.7; SD = 8.4 to Mean = 22.8; SD = 8.8; p = 0.11).											N/A	
**Diaz et al 2009 [42]**	Cuba	Case Study	Not mentioned	Infectious Diseases	Ecohealth approach used as a strategy to ensure active participation by the community, diverse sectors, and government. The approach allowed holistic problem analysis, priority setting, and administration of solutions.	Community Outcome	At the outset, 85% of the outbreaks of the dengue vector were in tanks located in the patios of the houses. Two years later only 29% were located in the patios. Currently, no outbreaks have been identified in the deposits located in the houses. It was found that 16% of the 4,878 courtyards in the territory were unhealthy. Two years after the end of the study, these constituted less than 1%; The number of unprotected tanks decreased from 62% to 8% (n = 4,678).											N/A	
**King et al** **2011 [55]**	American Samoa	Case Study	50 representatives from churches interviewed	Infectious Disease	Modified the initial Mass Drug Administration (MDA) strategy and partnered with various community groups including church groups for drug distribution, dissemination of messages about prevention of filariasis, and to encourage compliance. Developed radio and television ads to encourage "pill taking" and advertising locations of distribution.	Community Outcome	261 detailed surveys– 95.4% had heard of filariasis and increase (x2 = 19.2; p<0.001) from the 2003 KAP survey. Among those heard of filariasis 91.2% knew what it was an increase (x2 = 20.1; p<0.001) from 2003.											N/A	

Eight studies provided evidence on community outcomes in the form of increased community knowledge and awareness [15, 35, 43, 44, 49, 52, 53, 55]. Two studies involved interventions that focused on community health in general [15, 44], 1 on community mental health [43], 3 on infectious diseases [35, 52, 55], 1 on environmental health [53], and 1 on a healthy living intervention involving a physical activity trial [49]. Five studies reported on community outcomes relating to improved self-efficacy and confidence [22, 27, 46, 52, 54]. Two studies that reported on such outcomes had contextually tailored interventions on HIV and AIDS [52, 54]. Both studies reported positive impact on its target population including increased confidence and personal development among peer educators and sex workers, decreased HIV stigma, reduced proportion of men reporting that they had engaged in unprotected sex, and increased positive attitudes in condom use.

### Stakeholder perspectives

Study characteristics, along with the findings reported and the risk of bias assessments for studies that report on stakeholder perspectives can be found in Table 8 (See S1 File for table legend for risk of bias).

**Table 8:** Study characteristics, findings reported and the risk of bias assessments for studies that report on stakeholder perspectives.

**Study**	**Country**	**Study Design**	**Sample**	**Disease Category**	**Type of** **Community Involvement**	**Type of Outcome**	**Relevant Findings**							**Risk of bias**					
														**s**	**p**	**d**	**a**	**r**	**Overall**
**Abbema et al 2004 [56]**	The Netherlands	Intervention study	5000 residents in experimental areas, 7000 and 9500 in 2 control areas	Community Health	Intervention 'Arnhemse Broek, Healthy and Wellbeing'—direct involvement of community members during center visits for health priorities setting.	Stakeholder Perspectives	No significant effects on improved perceived health or health-related problems were found at the residents-level, and the problems identified. Results failed to prove effectiveness of the community intervention.												High
**Study**	**Country**	**Study Design**	**Sample**	**Disease Category**	**Type of** **Community Involvement**	**Type of Outcome**	**Relevant Findings**							**Risk of bias**					
														**s**	**d**	**n**	**c**	**Overall**	
**Cargo et al** **2011 [57]**	Canada	Cohort	28 at T1, 44 at T2, 51 at T3 (representatives from partners)	Community Health	University-Aboriginal community partnership for research.	Stakeholder Perspectives	1) Increased ownership of community program staff was perceived as primary owner at T1 and shared ownership with Community Advisory Board members at T2 and T3. 2) Trend tests indicated greater perceived ownership between T1 and T3 for CAB (p < .0001) and declining program staff (p < .001) ownership over time. 3) Academic partners were never perceived as primary owners.											Medium	
**Study**	**Country**	**Study Design**	**Sample**	**Disease Category**	**Type of** **Community Involvement**	**Type of Outcome**	**Relevant Findings**	**Risk of bias**											
								**1**	**2**	**3**	**4**	**5**	**6**	**7**	**8**	**9**		**Overall**	
**Ndirangu et al 2008 [58]**	United States	Qualitative	2 focus groups with 2 to 8 participants each from each of 3 communities	Community Health	Community-academic partnership. Members included a non-profit agency, university representatives, and participants from health, education, government, and lay leadership sectors.	Stakeholder Perspectives	1) Participants expressed satisfaction with the formation and maintenance of the committees and noted that the committees were still actively meeting in the community 2 years after they were formed. 2) Satisfaction with committee participation in community events. 3) Satisfaction with raising awareness about the committee in the community. 4) Participants spoke of individual benefits of becoming personally more aware of nutrition and physical activities.	Y	Y	Y	Y	Y	Y	Y	N	N	Y	Low (8/10)	
**Ferrera et al** **2014 [15]**	United States	Qualitative	23 youths interviewed	Community Health	CBPR used to form youth advisory board and youth involved in decision making and programming, as well as in a feedback and improvement role.	Stakeholder Perspectives	1) All youths (n = 23) had positive experiences with the program and believe it should be expanded to other schools.	Y	Y	Y	Y	Y	N	N	N	N	Y	Medium (6/10)	
**Heaton et al** **2014 [32]**	United States	Qualitative	Interviews, focus groups	Community Health	Collaborative partnership between 2 academic health centers and CBOs to determine topics, and develop a bi-directional educational seminar series called 'Community Grand Rounds' (CGR).	Stakeholder Perspectives	1) Good satisfaction with 'contract model' used to solidify partnership and lay out expectations. 2) CGR program met/exceeded their expectations.	N	Y	Y	Y	Y	Y	Y	Y	N	Y	High (8/10)	
**Derges et al** **2014 [59]**	United Kingdom	Qualitative	61 individuals interviewed	Healthy Living	Community Engagement Model—Well London program, community specific interventions for healthy eating, physical activity, and mental wellbeing delivered in socioeconomically deprived neighborhoods.	Stakeholder Perspectives	1) Positive benefits reported by those who participated in project activities. 2) Extent of benefits experienced was influenced by physical and social factors of each neighborhood. 3) Highest level of change in perception occurred in neighborhoods where there was social cohesion, personal and collective agency, and involvement and support of external organizations.	N	Y	N	Y	Y	Y	Y	N	N	Y	Medium (6/10)	
**Kennedy et al 2010 [60]**	United Kingdom	Qualitative	35 key informants interviewed	Healthy Living	‘Lay food and health workers’ and professionals involved in delivering local food and health initiatives in less-affluent neighborhoods.	Stakeholder Perspectives	1) Salient benefits identified were increased service coverage, ability to reach the "hard to reach", as well as personal development and enhanced social support.	Y	Y	Y	Y	Y	Y	Y	N	Y	Y	Low (9/10)	
**Study**	**Country**	**Study Design**	**Sample**	**Disease Category**	**Type of** **Community Involvement**	**Type of Outcome**	**Relevant Findings**											**Risk of Bias**	
**Mason et al** **2014 [61]**	United States	Case Study	10 parks	Healthy Living	A CBPR evaluation engaged community and academic partners done to evaluate the acceptability, sales impact, and implementation barriers for the Chicago Park District's 100% Healthier Snack Vending Initiative aimed at strengthening healthful vending efforts.	Stakeholder Perspectives	1) Staff (100%) and patrons (88%) reacted positively to the initiative. 2) Patrons overwhelmingly approved of the more healthful snack vending items—88% reported liking the snack vending items they tried, 98% indicated that would purchase the snacks again. 3) Sales exceeded the expectations of both district staff and vendors. Average monthly sales volume per machine also exceeded industry sales estimates of $300 per month for snack vending machines located in “average” locations, which typically have 10 sales per day.											N/A	
**Basu Roy et al 2014 [62]**	United States	Case Study	69 participants interviewed, 4 focus groups	Non Communicable Diseases	Queens Library HealthLink program, a CBPR academic–community partnership, aimed to reduce cancer disparities through neighborhood groups, Cancer Action Councils that convened in public libraries.	Stakeholder Perspectives	1) 78% of 69 survey participants agreed that community interests are well represented in council projects. 2) 97% agreed that council members have a voice in the development of programs. 3) 97% acquired useful knowledge about programs, services, or people in the community. 4) 94% developed valuable relationships. 5) 94% reported increased ability to contribute to communities. 6) 91% felt they made a greater impact than they would have on their own. 7) 88% developed an enhanced ability to address an important issue. 8) Participants reported accomplishments in planning and hosting of events, cancer screenings, and conducting health fairs.											N/A	

In five studies, participants reported positive experiences or satisfaction with the community participatory initiative [15, 58, 59, 61, 62], three of which involved community-academic partnerships [58, 61, 62]. Six studies reported on stakeholder perspectives that reflected positive community-level outcomes [57–59, 61–63]. Two of these studies reported greater awareness of the targeted health issue or services among the community, both of which involved community-academic partnerships [59, 62]. Three studies reported perceptions relating to the processes of involving the community, although results were mixed [44, 57, 58]. Two of the studies reported stakeholder satisfaction with service coverage, staff development, enhanced networks, and creation of new alliances [44, 58]. However, another qualitative study that investigated perspectives of a health impact assessment among native participants reported otherwise, highlighting the need to account for a community’s history of colonization and forced assimilation in the community engagement process [57]. At a more fundamental level, community participation has been perceived to have facilitated community ownership and development as reported in two studies [57, 62].

### Empowerment

Study characteristics, along with the findings reported and the risk of bias assessments for studies that report on empowerment can be found in Table 9 (See S1 File for table legend for risk of bias).

**Table 9:** Study characteristics, findings reported and the risk of bias assessments for studies that report on empowerment (n = 7).

**Study**	Country	Study Design	Sample	Disease Category	Type of Community Involvement	Type of Outcome	Relevant Findings	Risk of bias										
								**1**	**2**	**3**	**4**	**5**	**6**	**7**	**8**	**9**		**Overall**
**Gibbons et al 2016 [28]**	United States	Qualitative	3 focus groups, 8 in-depth interviews, 31 individuals surveyed	Community Health	Community-academic collaboration 'Community Health Initiative: Creating a Healthier East Baltimore Together' using CBPR.	Empowerment	Community participation led to empowerment of residents, through skills based training as part of the asset mapping research process.	N	Y	Y	Y	Y	N	Y	N	N	N	Medium (5/10)
**Trettin et al 2000 [29]**	United States	Qualitative	6 to 14 participants of 3 focus groups (total n = 60)	Community Health	Volunteer-based community health advisory program developed to increase residents' access to health services, stimulate their interest in health, disease prevention, and awareness of health-related environmental issues, and empower residents to be more involved in community health.	Empowerment	Sense of empowerment fostered among participants when they were given greater control over the direction of the program.	N	Y	Y	Y	Y	Y	Y	N	N	Y	Medium (7/10)
**Ferrera et al 2014 [15]**	United States	Qualitative	23 youths interviewed	Community Health	CBPR used to form youth advisory board and youth involved in decision making and programming, as well as in a feedback and improvement role.	Empowerment	Improved sense of agency amongst students. Community participation facilitated an understanding of how students may have a positive impact on their community. "Individual levels of empowerment" described in terms of youth's ability to "reach out" and disseminate health information to their family members and the immigrant community. Reaching out to and advocating for undocumented immigrants helped them to gain confidence and knowledge on accessing services. They felt empowered to motivate others to do the same.	Y	Y	Y	Y	Y	N	N	N	N	Y	Medium (6/10)
**Kennedy et al 2010 [60]**	United Kingdom	Qualitative	35 key informants interviewed	Healthy Living	‘Lay food and health workers’ and professionals involved in delivering local food and health initiatives in less-affluent neighborhoods.	Empowerment	Empowerment was perceived as both an individual benefit and a benefit to the community resulting from the program.	Y	Y	Y	Y	Y	Y	Y	N	Y	Y	Low (9/10)
**Study**	**Country**	**Study Design**	**Sample**	**Disease Category**	**Type of** **Community Involvement**	**Type of Outcome**	**Relevant Findings**											**Risk of Bias**
**Setti et al** **2010 [53]**	Brazil	Case Study	24 participants	Environmental Health	The Neighborhood Ecological Program that involved the participation and empowerment of citizens in health promotion and sustainable development.	Empowerment	Participation in the implementation of the program favored empowerment among individuals and groups.											N/A
**Wilson et al 2014 [54]**	United States	Case Study	71 participants	Infectious Diseases	CBPR used to develop the ‘Barbershop Talk With Brothers’ program—a community-based HIV prevention program that seeks to improve individual skills and motivation to decrease sexual risk, and that builds men’s interest in and capacity for improving their community’s health.	Empowerment	Increased perceptions of community empowerment (Mean = 18.7; SD = 4.0 to Mean = 19.6; SD = 3.4; p = 0.06).											N/A
**Diaz et al 2009 [42]**	Cuba	Case Study	Not mentioned	Infectious Diseases	Ecohealth approach used as a strategy to ensure active participation by the community, diverse sectors, and government. The approach allowed holistic problem analysis, priority setting, and administration of solutions.	Empowerment	Community was strengthened and empowered by creating neighborhood groups, and by developing communication skills to work in such programme.											N/A

Three studies described how participation in a community initiative fostered engagement [28, 42, 53]. Two studies described how greater agency, i.e. the capacity of individuals to act on their own accord, interacted with empowerment [15, 29]. One study involved a volunteer-based community health advisory program that sought to increase access to health services which reported a sense of empowerment among participants after they were given greater control over program direction [29]. The other study, involving a youth advisory board formed through CBPR, reported an improved sense of agency amongst students [15]. One study described specifically how gaining skills through participation led to empowerment. The study involved a community-academic collaboration that led to resident empowerment through skills based training that was included in the CBPR research process[28]. In another study on active participation strategies for environmental solutions, community groups were reportedly mobilized to make changes in their own community, resulting in the strengthening and empowerment of the community [42].

### Health outcomes

Study characteristics, along with the findings reported and the risk of bias assessments for studies that report on health outcomes can be found in Table 10 (See S1 File for table legend for risk of bias).

**Table 10:** Study characteristics, findings reported and the risk of bias assessments for studies that report on health outcomes (n = 12).

**Study**	**Country**	**Study Design**	**Sample**	**Disease Category**	**Type of** **Community Involvement**	**Type of Outcome**	**Relevant Findings**	**Risk of bias**					
								**s**	**p**	**d**	**a**	**r**	**Overall**
**Solomon et al 2014 [49]**	United Kingdom	RCT (Stepped wedge cluster)	10,412 adults (intervention = 4693; control = 5719)	Healthy Living	Intervention developed with local partners using local knowledge and resources to facilitate local involvement in planning, promotion, and delivery of a physical activity intervention.	Health Outcome	1) Intervention did not increase the odds of adults meeting the physical activity guidelines (adjusted OR 1.02, 95% CI: 0.88 to 1.17; P = 0.80). 2) Weak evidence of an increase in minutes of moderate-and-vigorous-intensity activity per week (adjusted mean difference = 171, 95% CI: -16 to 358; P = 0.07).						High
**Caprara et al** **2015 [17]**	Brazil	RCT	10 intervention clusters, 10 control clusters	Infectious Disease	Intervention adopted an eco-health approach to involve community through workshops, clean-up campaigns, mobilization of school children and seniors, and distribution of information, education and communication materials.	Health Outcome	1) Impact on vector densities—overall vector density increased from dry season (pre-intervention) to the rainy season (post-intervention) as expected, but the increase was significantly higher in the control area (p-values: House Index = 0.029; Container Index = 0.020; Breteau Index = 0.014, Pupae per person = 0.023) demonstrating the protective efficacy of the intervention.						Low
**Study**	**Country**	**Study Design**	**Sample**	**Disease Category**	**Type of** **Community Involvement**	**Type of Outcome**	**Relevant Findings**	**Risk of bias**					
								**s**	**p**	**d**	**a**	**r**	**Overall**
**Sansiritaweesook et al 2015 [16]**	Thailand	Intervention study	182 informants, 562 surveillance networks, 21,234 villagers	Environmental Health	7-step process used to develop a model for local drowning surveillance system based on community participation.	Health Outcome	1) In the year after system implementation the non-fatality drowning rate in target areas fell to zero, the non-fatality rate in control areas increased. 2) Fatality rate in target areas dropped to 4.5 per 100,000 but remained the same in control areas. Incidence rate ratio of injuries in the comparison areas was 23.32 times higher than in the target areas (95% CI: 3.081–176.599, p = 0.002).						Medium
**Hoelscher et al 2010 [20]**	United States	Intervention study	15 schools receive BPC intervention, matched with 15 schools that receive BP only	Healthy Living	School-based obesity prevention program (CATCH BP) versus complimentary program (CATCH BPC) that formed partnerships with external community organizations.	Health Outcome	1) In terms of percentage of students classified overweight or obese, CATCH BP had a decrease of 1.3 points (3.1%) (P = 0.33) while CATCH BPC had a decrease of 8.3 points (8.2%) (P<0.005).						Unclear
**Sharpe et al** **2011 [64]**	Canada	Intervention Study	40 after-school program sites [6 BGC CKC sites, 12 comparison sites]	Healthy Living	CATCH Kids Club (CKC) program integrated into the programming of 2 agencies–the YMCA and the Boys and Girls Clubs (BGC).	Health Outcome	1) Nearly all sites, with the exception of the BCG baseline program (a sports program) achieved greater than 50% of time spent in moderate to vigorous physical activity (MVPA). 2) Significant differences were not found between levels of MVPA at intervention and comparison sites (59.3% vs. 64.2%) or at intervention sites at baseline vs. post intervention (59.3% vs. 52.1%). 3) BCG sites had significantly higher levels MVPA in CKC programs than in sports programs (70.8% vs. 35.2%).						Unclear
**Clark et al** **2014 [22]**	United States	Intervention study	1,477 parents of children with asthma in coalition target areas and comparison areas	Non Communicable Disease	Allies Against Asthma program—a 5-year collaborative effort by 7 community coalitions designed to change policies regarding asthma management in low-income communities of color.	Health Outcome	1) At follow-up, Allies children experienced significantly fewer daytime symptoms than did comparison children over the preceding 2 weeks (3.03 vs. 3.91; p = 0.008). 2) Annual differences in daytime symptoms were not evident. 3) Night time symptoms over the preceding 2 weeks (2.35 vs. 3.41; p = 0.004) and 1 year (55.17 vs. 81.45; p = 0.003) were significantly less frequent among Allies children than among comparison children. 4) 29% of Allies children went from experiencing some symptoms at baseline, to experiencing no symptoms at follow-up. In comparison group, 19% of children became symptom free. 5) After adjustment for race/ethnicity, age, gender, and community site, the Allies children had 2 times the odds of comparison group of moving from some symptoms at base-line to none at follow-up (odds ratio = 1.9; 95% CI = 1.17, 2.96).						Unclear
**Clark et al 2013 [65]**	United States	Intervention study	12,361 in intervention group, 14,475 in comparison group	Non Communicable Disease	6 Allies Against Asthma coalitions mobilized stakeholders for policy change in asthma control.	Health Outcome	1) Allies Children were significantly less likely (p<0.04) to have an asthma related hospitalization, and less likely (p<0.02) to have such healthcare use. 2) The hazard of having a hospitalization, ED, or urgent care visit at any time during the 5-year time period was 6% to 7% (p<0.01 and p<0.02) greater for children in the comparison group than those in the Allies communities.						Medium
**Study**	**Country**	**Study Design**	**Sample**	**Disease Category**	**Type of** **Community Involvement**	**Type of Outcome**	**Relevant Findings**	**Risk of bias**					
								**s**	**d**	**n**	**c**	**Overall**	
**Davison et al** **2013 [46]**	United States	Cohort	423 children age 2–5	Healthy Living	CBPR used to develop and pilot test a family-centered intervention for low-income families with preschool-aged children.	Health Outcome	1) Compared with pre-intervention, children at post intervention exhibited significant improvements in their rate of obesity, light physical activity, daily TV viewing, and dietary intake (energy and macronutrient intake). 2) Positive trends observed for BMI z score, sedentary activity and moderate activity.					Low	
**Reeve et al** **2015 [25]**	Australia	Cohort	N/A	Non Communicable Diseases	A health service partnership between an Aboriginal community-controlled health service, a hospital, and a community health service that implemented an integration of health promotion, health assessments, and chronic disease management.	Health Outcome	Long-term outcomes– 1) Decreased number of deaths and emergency admissions. 2) Increased screening for alcohol and tobacco use.					Medium	
**Oba et al** **2011 [66]**	Thailand	Cohort	160 pre-diabetes patients	Non Communicable diseases	Community participation in 5 processes of the assessment, diagnosis, planning, implementation, and evaluation of a diabetes health promotion program in a primary care unit.	Health Outcome	1) After intervention, the mean score for exercise activity among the persons with pre-diabetes was significantly higher (before 2.72 +/- 1.24 SD; after 3.00 +/- 0.980 SD; paired t-test -2.95; p = 0.004). 2) The mean score for BMI was lower after intervention (before 24.83 +/- 4.47 SD; after 24.38 +/- 4.330; paired t-test 4.77; p = 0.001). 3) The mean score for waist circumference was lower after intervention (before 83.34 +/- 9.12 SD; after 81.66 +/- 8.830; paired t-test -2.95; p = 0.004). 4) The mean score for systolic blood pressure was lower after intervention (before 128.45 +/- 13.94; after 125.84 +/- 10.632; paired t-test 2.67; p = 0.008). Overall, this meant that community participation provided proactive services to persons with pre-diabetes.					Medium	
**Study**	**Country**	**Study Design**	**Sample**	**Disease Category**	**Type of** **Community Involvement**	**Type of Outcome**	**Relevant Findings**					**Risk of Bias**	
**Barnes et al** **2006 [43]**	United Kingdom	Case Study	Not mentioned	Non Communicable Diseases	Users of a community mental health inter-professional training program (partnerships with service users) involved in the commissioning, management, delivery, participation, and evaluation of the program, as trainers and as course members.	Health Outcome	1) The service users with whom the students worked (n = 72) improved significantly over 6 months in terms of their social functioning [F (1,62) = 4.12, p = 0.047] and life satisfaction [F (1,59) = 6.43, p = 0.014], but not in their mental health status [F (1,65) = 0.85, p = 0.352]. 2) Users in the comparator groups also improved in life satisfaction and social functioning, but the improvement in social functioning was significantly greater for those users in the program group than for the comparators [F (3,155) = 7.31, p< 0.001].					N/A	
**King et al** **2011 [55]**	American Samoa	Case Study	50 representatives from churches interviewed	Infectious Disease	Modified the initial Mass Drug Administration (MDA) strategy and partnered with various community groups including church groups for drug distribution, dissemination of messages about prevention of LF, and to encourage compliance. Developed radio and television ads to encourage "pill taking" and advertising locations of distribution.	Health Outcome	1) After the MDA program change coverage increased from 49% to 71% and remained high in subsequent years. Reported compliance for people living in surveyed households was 86.4% (95%CI, 83.8–88.9%). 2) 94.6% of respondents reported taking tablets at least once since program inception, 73.6% reported taking tablets every MDA and 81.6% reported taking tablets during the last MDA (2004); among those who took tablets in 2004, 82.6% received prior notification, an improvement from 2003 (x2 = 7.4; p<0.01).					N/A	

The health impact of community participation interventions was the most evident among studies involving non-communicable diseases. All five studies reported positive health outcomes including decreased hospital admissions [25, 65], reduced clinical symptoms [22], improved behavioral risk factors such as exercise [46, 49, 64, 66], improved quality of life[43], and decreased mortality over time [16]. Two studies on infectious diseases reported positive health outcomes in terms of greater community compliance to the prevention and treatment of lymphatic filariasis which was the targeted disease of the community participation program [55], and a lower rate of increased vector density of a dengue control intervention[17]. Two out of 4 studies relating to healthy living reported positive results relating to improvements in obesity rates [20, 46], while the other 2 studies targeting physical activity did not find these interventions effective in promoting health outcomes [49, 64]. Only one study on environmental health reported on health outcomes where the implementation of the local drowning surveillance system resulted in reductions in non-fatal drowning rates, drowning fatality rates and incidence rate ratios of injuries [16].

## Discussion

This review explores reported outcomes of community involvement and participation and presents a conceptual model to frame these outcomes, beginning with a foundation of process outcomes and community outcomes as necessary to achieving robust health outcomes, while recognizing the influence of stakeholder perspectives and empowerment.

Our review highlights the importance of both process and outcomes evaluations when assessing community involvement interventions. Process outcomes, especially those that reflect on organizational processes, are the results of intra- and inter- organizational negotiating and learning, that over time results in “trust” and “authentic” relationships which ultimately drive partnerships forward [66]. Few studies report on the community processes that result from these initiatives, such as increased outreach, volunteerism or other “conversion” of community members into active members. From an organizational perspective, many studies reported on the learning phases wherein organizational relationships are established and built. Partnerships in this phase mostly report process outcomes as they learn ways of working both together and with the community [43]. This learning curve is important in developing contextually appropriate interventions and those studies that invest in this stage report success in program development and implementation [25].

Failing to account for contextual learning can result in failure to work together to achieve goals, and this is especially important in vulnerable populations and those communities with a history of colonization and forced assimilation [55]. This speaks to the international Aboriginal self-determination movement which calls for program development for indigenous people by indigenous people that integrates underlying theoretical and cultural frameworks into applied public health [17]. Past research has shown how community participation interventions have been viewed as an initiative to improve health outcomes rather than a process to implement and support health program to sustain these outcomes [20, 46]. However, our findings highlight that examining community participation as a “process” is equally as important, and furthers the understanding that outcomes could be influenced by shifts in social, economic, and political contexts over time.

Overall, community-level outcomes were the most common measure reported across the studies. Findings from our review demonstrate that successful community outcomes were most evident among interventions that included outreach activities such as: health camps, community fairs, and partnerships with schools and religious groups [49, 64]; targeted interventions that delivered tailored and specific health knowledge [16]; and interventions that encouraged relationship building with the wider community [28, 41, 44]. CBPR was also beneficial in developing trust between community and academic partners through the creation of a level-playing environment where members could decide on health priorities collectively [28, 29, 67]. In another review that examined the effectiveness of community engagement in health intervention planning and delivery, community participation initiatives were reportedly linked to positive gains in social capital, social cohesion, and in capacity building among the community [16, 22]. Furthermore, a systematic review addressing what indigineous Australian clients valued about primary health identified how community participation influences access, acceptability, availability, responsiveness and quality of services, with the potential of increasing utilisation and ultimately improving health outcomes [68]. Another study also identified how increased community participation could also address the social determinants of health outcomes through increased local or Indigenous employment services [69]. In our review however, very few studies reported on such community outcomes, which are inherently more difficult to define and measure given its subjectivity.

In terms of population level outcomes, our findings indicate that there is a problematic reliance on empowerment as an outcome measure of community participation interventions. Some studies report on community empowerment and empowering of participants as a community level improvement resulting from participation in a community project or initiative [67]. Empowerment is perceived as beneficial and a positive outcome of community participation, often constructed through qualitative exploration of participants and residents’ perceptions, but without a robust definition and measurement of impact, caution is required in attributing the outcomes reported to actual community empowerment. Furthermore, care must be taken not to reduce empowerment to a component of a bureaucratic process while conflating these debatable definitions and measures of empowerment to represent tangible power and influence [70]. Empowerment as an outcome requires sustained community engagement, which is dependent on program sustainability. While there may be many barriers to sustainability, the greatest challenges can be political [71].

Findings from our review indicate that the ultimate aim for most community involvement programs is to improve health and wellbeing of a particular community; however, indicators were difficult to obtain and measure. Changes in health status usually require long-term monitoring and may not be measurable over a single program cycle. In our review, health outcomes are most commonly reported for community involvement interventions addressing non-communicable diseases and healthy living, and findings presented are generally mixed. For instance, some healthy living interventions reported no significant effect of physical activity interventions on health outcomes [15, 17, 24, 46, 55, 57] while others reported the contrary [22, 65]. Nonetheless, interventions that are contextually targeted which have specific goals at the outset that are monitored over time seem to have greater success in achieving positive health outcomes [16, 44, 54]. As highlighted in other reviews, identifying that a positive outcome or change is specifically attributable to community participation is a complex task [44]. Community participation initiatives usually do not happen as a direct and linear intervention to improve health, but rather consists of complex processes and interactions [7]. Our review reports promising evidence that community engagement has a positive impact on health, especially when supported by a strong organizational and community foundation.

Despite the variability in interventions, there are some positive community participation examples that provide convincing evidence of benefits as demonstrated by the six RCTs identified in this review, two of which were of high quality given its overall low risk of bias [17–19, 48–50]. Boivin’s study elucidates that community involvement is central to setting priorities in driving healthcare improvement at the population level [19] while Caprara’s study presents social participation as an effective tool in facilitating environmental management for improved dengue vector control [17]. It should be noted however, that all studies described were context specific, hence the external validity of these studies are inevitably limited. Ultimately, there is ‘no one size fits all’ approach to community participation that will ensure intended positive outcomes and community participation that is tailored to context is fundamental in ensuring the provision of equitable health care and optimization of interventions to improve health [64].

### Strengths and limitations

This systematic review on outcomes of community participation in high and upper middle income countries is the first of its kind to be conducted. A strength of this review was the use of a wide range of databases and the inclusion of papers in multiple languages to ensure broad representation. However, majority of the studies identified were conducted in the United States which could be a result of publication bias. It is highly likely that not many real world community participatory initiatives are evaluated robustly according to epidemiological standards, and it is possible that studies with null findings are less likely to be published. Additionally, given the broad scope of our inclusion criteria, the search produced a large amount of literature on community participation for eligibility assessment and synthesis. Nevertheless, prioritizing studies that had the best quality evidence in outcomes reported allowed for the data extraction and synthesis process, and the risk of bias assessment, to be done comprehensively and with rigour.

#### Implications for research

Our review shows that while community participation and involvement is well documented from a case study and qualitative perspective, there is a need for more robust program evaluations and studies that measure and report long-term outcomes. Studies were largely descriptive or only had a evaluative component as part of a case study. While descriptive reports provide insight into program successes and operationalisation they would benefit from more robust methodology and reporting to determine stronger causal linkages between intervention components and desired outcomes.

Our review included six RCT studies that serve as positive examples for evaluating community participation programs. However, it must be noted that while RCTs are considered the gold standard in research methodology; difficulties in applying experimental designs at the population level is evident and well documented [7]. A particular challenge will be to account for the multi-faceted health and social dimensions of community participation in drawing definitive linkages and pathways that explain how community participation leads to a desired community or health outcome[6].

Importantly, no studies reported on outcomes relating to costs. Further evaluations are needed to examine the cost-effectiveness of real-world interventions and draw comparisons between the varying approaches of community participation and involvement. Such research is imperative to support evidence-based policy-making by identifying community participation programs that can achieve the greatest health return on investment.

### Implications for policy

Evidence garnered from this systematic review presents some of the successes of community participation in yielding positive outcomes at the organizational, community, and individual level in high and middle-income countries. It is a worthwhile endeavour for policymakers to devote resources in enabling community engagement, creating platforms for involvement, and in facilitating successful collaborations or partnerships within the health sector and beyond. Nonetheless, addressing issues of power relations, developing trust with the community, and understanding the political, social, and economic contexts in which initiatives are supported, is imperative in any form of community engagement effort.

Based on the findings of this review, we have developed a new outcomes framework for community participation which policy-makers can utilise to prioritise program outcomes and justify resource allocation in program design and implementation. Consideration of the interplay of social and cultural factors is essential when exploring perspectives of community members on outputs of such initiatives, while empowerment and power relations are key elements that should be taken into account with more robust measurements. As policy-makers consider new and effective ways of planning, implementing, monitoring, and evaluating community involvement programs, the evidence here can contribute in providing some clarity to the process and supporting the development of evidence based policies.

### Conclusion

Community participation is a fundamental element of an equitable and rights-based approach to health that is proven effective in optimizing health interventions for positive public health impact. This review adds to this evidence base supporting the utility of community participation in yielding positive outcomes at the organizational, community, and individual level across a wide range of health domains. Our findings present process and community outcomes as necessary to achieving robust health outcomes. This supports the notion that participatory approaches and health improvements do not happen as a linear progression, but rather consists of complex processes influenced by an array of contextual factors. Overall, it is evident that community involvement is key in priority setting to drive healthcare improvement and that interventions utilizing community involvement can benefit from a contextualizing learning phase whereby organizational relationships and trust can develop. Our review highlights the need for more robust program evaluations of community participation initiatives that measure long-term outcomes and cost-effectiveness, in more settings globally.

## Supporting information

S1 Table PRISMA checklist. (DOCX)

S1 File Legend for outcome tables. (DOCX)
