# Supplementary material for: Community participation in health services development, implementation, and evaluation: A systematic review of empowerment, health, community, and process outcomes
Source: PLoS One. 2019 May 10;14(5):e0216112. doi: 10.1371/journal.pone.0216112 (PMC6510456; doi:10.1371/journal.pone.0216112)
Supplement: S1 File — (DOCX) [file pone.0216112.s002.docx]

**S1 File: Legend for outcome tables**

|  | High risk of bias |
| --- | --- |
|  | Unclear risk of bias |
|  | Low risk of bias |

ROB category for RCTs and intervention study

s – selection bias

p – performance bias

d – detection bias

a – attrition bias

r – reporting bias

ROB category for observational studies

s – selection bias

d – differential misclassification

n – non-differential misclassification

c – confounding

| Y | Yes |
| --- | --- |
| N | No |

ROB category for qualitative studies

1 – Background

2 – Aims

3 – Context

4 – Sampling

5 – Data collection and analysis

6 – Reliability of data analysis

7 – Clarity about conclusion

8 – Reflexivity

9 – Generalisability

10 - Ethics
